# Sensory and Physicochemical Quality Characteristics of Haddock Fish Cake Enriched with Atlantic Mackerel Quality of Fish Cakes

**DOI:** 10.17113/ftb.59.01.21.6695

**Published:** 2021-03

**Authors:** Janna Cropotova, Revilija Mozuraityte, Inger Beate Standal, Olga Szulecka, Tomasz Kulikowski, Adam Mytlewski, Turid Rustad

**Affiliations:** 1 Department of Biotechnology and Food Science, Norwegian University of Science and Technology, Sem Saelands vei 6/8, 7491 Trondheim, Norway; 2 SINTEF Ocean, Brattorkaia 17C, 7010 Trondheim, Norway; 3 National Marine Fisheries Research Institute, Kollataja 1, 81-061 Gdynia, Poland

**Keywords:** fish cake, haddock, Atlantic mackerel, sensory attributes, quality parameters, nucleotides, NMR metabolomics

## Abstract

**Research background:**

It is desirable to increase the consumption of pelagic fish rich in long-chain omega-3 fatty acids. Partial replacement of traditionally used white fish species by pelagic fish will increase the content of omega-3 fatty acids, and thus improve the nutritional value but it may also affect the consumer acceptance. The aim of this study is to assess the physicochemical and sensory quality of novel fish cake prototypes prepared from haddock and mackerel mince.

**Experimental approach:**

Fillets of haddock and Atlantic mackerel were used as raw material for preparation of fish cakes. The fish fillets were minced, mixed together (in haddock/mackerel mass ratio of 100:0, 75:25 and 50:50%) with salt, potato starch, pepper and full fat milk. Physicochemical and sensory analyses were further performed.

**Results and conclusions:**

The fatty acid composition analysis showed that the recommended daily intake of 250 mg of eicosapentaenoic acid and docosahexaenoic acid can easily be reached by consumption of fish cakes enriched with mackerel. The oxidation levels of all fish cakes were low in terms of peroxide value and thiobarbituric acid reactive substance assay (TBARS). Fish cakes prepared with higher mass fraction of mackerel mince (>50%) had significantly (p<0.05) softer texture than other fish cakes due to higher amount of fat in their formulations. At the same time, these fish cakes were significantly darker than haddock-based (>50%) fish cakes due to higher myoglobin content in the fish muscle. Moreover, fish cakes with higher amount of mackerel mince had increased yellowness due to the accumulation of water-soluble (r=0.990, p<0.05) and fat-soluble (r=0.976, p<0.05) TBARS. Metabolites relevant for taste and quality were quantified by using ^1^H nuclear magnetic resonance (NMR) spectroscopy. The mass fraction of anserine, trimethylamine oxide and β-alanine decreased, while the mass fraction of histidine, glutamic acid and alanine increased with the addition of mackerel. Sensory tests have shown the addition of mackerel did not reduce consumer acceptability towards the new fish cakes.

**Novelty and scientific contribution:**

The research demonstrated that Atlantic mackerel can be successfully used for partial replacement of white fish species in fish cake formulations to produce healthy and tasty ready-to-cook products and increase the consumption of small pelagic fish in Europe.

## INTRODUCTION

Consumer awareness of health benefits associated with seafood consumption has increased the demand for fish products over the last decades (*1*). Fish is a valuable source of essential nutrients and bioactive compounds such as long-chain omega-3 fatty acids – docosahexaenoic acid (DHA) and eicosapentaenoic acid (EPA), fat-soluble vitamins (E and D), low-molecular-mass metabolites, such as anserine and taurine, and easily digestible proteins (*1*-*4*).

A significant global increase in fish consumption has enhanced people’s diets all over the world, making them more nutritious (*5*). In 2017, fish accounted for about 19% of the global population’s intake of animal protein (*6*). The European fish consumption has grown continuously during the last few decades, and currently 42% of European consumers eat fish and seafood products at least once a week (*5*). There are, however, differences among countries. Norway has a relatively high fish consumption compared to EU-countries, but it is decreasing. The results from household panel surveys show a reduction of 7% from 2016 to 2017, while from 2012-2017 the total reduction was 17% (*7*). The reduction is largest among people aged between 18 and 34, and one reason is that younger people do not have the same traditions in preparing fish as the older population (*7*). In addition, the consumption of pelagic fish species is much lower than the consumption of other fish species such as salmon or cod, although the total catch of pelagic fish has increased gradually during the last years in the EU and Norway (*5*). This situation can partially be explained by a lack of diversity of processed pelagic fish products (*8*), and lack of promotion towards young consumers. Traditionally, small pelagic fish have been used in the manufacture of canned fish products such as mackerel in oil or tomato sauce, salted herring, canned sardines, *etc*. To increase the consumption of pelagic fish, and thereby improve the diet of the population, there is a need to increase the diversification of fish products in Europe. Ready-to-eat/ready-to-cook fish products, *i.e.* canned fish, fish cakes rich in omega-3 fatty acids, vitamins and natural antioxidants could be an option for a healthy diet.

The consumers readiness to taste new fish products displayed by European Market Observatory for Fisheries and Aquaculture Products (EUMOFA) (*5*) demonstrates the importance of having a diverse range of fish products obtained from different fish species. Moreover, a stressed lifestyle affects dietary patterns, increasing intake of processed and fast food. Increasing the availability of easy to prepare healthy and tasty food such as canned fish, fish cakes, fish balls, fish burgers, *etc*. with maximal use of pelagic fish species such as Atlantic mackerel, and studying consumer acceptance of the developed products is a highly relevant approach. To study consumer acceptance, sensory analysis is an important step for assessing taste preferences of consumers. A sustainable development of the fish processing sector in Europe can reduce the import and re-import of fish products (*e.g.* fish caught in the EU that is processed in Asia and imported back for consumption in the EU countries) through sustainable use of resources (*9*).

Fish cakes, *i.e.* a formulated fish product typically consisting of 50-80% fish and prepared by frying in a pan or oven have traditionally been consumed in many European countries, including Norway and Poland. In the Scandinavian countries, they are generally made from white fish species such as cod or haddock, with the addition of potato starch, milk, herbs and salt. At the same time, fish cakes made from fatty fish such as salmon and shellfish have recently become popular in Norway and Asian countries such as Thailand, Japan or India (*10*).

Atlantic mackerel is one of the main fish species used in production of canned fishery products in Norway and Poland, ranking among the top small pelagic commodity groups both in volume and value in 2017 and 2018 in Europe (*5*, *6*). Nevertheless, although pelagic fish have been recognized as a valuable component of the diet in many European countries including Poland and Norway for many years, their market position is low compared to other fish species. There is a potential of increasing both the consumption and the value of mackerel by producing higher value products than the traditional canned products (*11*). In addition, pelagic fish has not been used for production of fish cakes to any large degree neither in Norway nor in the EU.

The aim of the present study is to develop novel fish cake formulations enriched with Atlantic mackerel raw material rich in essential omega-3 fatty acids and to evaluate the physicochemical and sensory characteristics of the developed products. This will provide valuable data for both food manufacturers and professionals on quality characteristics and consumer acceptance and attitudes towards new fish cakes based on haddock and Atlantic mackerel.

## MATERIALS AND METHODS

### Preparation of fish cakes

Fillets of haddock (*Melanogrammus aeglefinus*) and Atlantic mackerel (*Scomber scombrus*) used as raw material for preparation of fish cakes, were purchased fresh from a local retailer (Ravnkloa, Trondheim, Norway). Prior to mincing, the fillets were kept on ice. Other commercial ingredients such as salt, potato starch, pepper, full cream milk and rapeseed oil were bought in a local supermarket Rema 1000 (Trondheim, Norway).

To make fish mince, pieces of mackerel and haddock (1470 g, (4±1) °C) were mixed together in haddock/mackerel mass ratio of 100:0, 75:25 and 50:50%, and further mixed with 30 g salt for 30 s in a food processor (Bosch Universal Plus, Gerlingen, Germany). Potato starch (50 g) and pepper (2 g) were added and mixed for 15 s, followed by the addition of 440 g full fat milk and mixing for 40 s. Fish cakes of 60 g were made from the mixture and fried in rapeseed oil (three tablespoons in a frying pan) for 3 min on each side. After frying, the cakes were transferred to the kitchen oven to cook until the inside temperature reached 75 °C. The cakes were cooled down, vacuum packed and stored at 4 °C for two days before the consumption and analysis. Physicochemical analyses of fish cakes were performed on the third day after preparation.

### Extraction of lipids and oxidation products

Lipids were extracted from the fish fillets and fish cakes by the Bligh and Dyer method (*12*), which uses a binary mixture of chloroform (Sigma-Aldrich, Merck, Oslo, Norway) and methanol (purity 99.9%) in a volume ratio 1:1, diluted with distilled water (3:4) as an extraction medium. The extraction was performed in duplicate. The total amount of lipids was determined gravimetrically by placing the aliquot of the chloroform phase extract in the pre-weighed glass tube kept in a heating block at 60 °C under a stream of nitrogen until evaporating to dryness. Lipid extracts in chloroform phase and water/methanol phase were stored at -80 ºC prior to analysis of peroxide value and thiobarbituric acid reactive substances.

### Lipid oxidation products

After lipid extraction by Bligh and Dyer method (*12*), determination of fat- and water-soluble secondary lipid oxidation products such as aldehydes was performed in the chloroform and water/methanol phases, respectively.

Thiobarbituric acid reactive substances (TBARS) in chloroform phase were determined as described by Ke and Wooyewoda (*13*). As a standard 1,1,3,3-tetraethoxypropane (Sigma-Aldrich, Merck, Oslo, Norway) was used. The results were expressed in mg malondialdehyde (MDA) per kg sample±standard deviation of at least four parallels.

TBARS in methanol/water phase were determined as described by Schmedes and Holmer (*14*) using 1,1,3,3-tetraethoxypropane (Sigma-Aldrich, Merck) as a standard. The results are expressed in mg MDA per kg sample±standard deviation of at least four parallels.

Peroxide value (PV) was measured in the lipid extract using the modified Shantana and Decker (*15*) method as described by Baron *et al*. (*16*). A volume of 1 mL aliquot of total lipid extract was mixed with 10 mL chloroform/methanol (7:3, *V/V*), then thiocyanate solution (50 μL, 30%) was added and the reaction mixture was left to react for 5 min at room temperature. The absorbance was read at 500 nm using a spectrophotometer (UV mini 1240; Shimadzu Corp., Tokyo, Japan). PV was determined using a standard curve with FeCl_3_ as standard. Analysis was performed in triplicate. The results were expressed in meq peroxide per kg lipids as sample±standard deviation.

### Determination of fatty acid composition

The fatty acid (FA) profile of the extracted lipids in chloroform phase was determined using an Agilent Technologies 7890A (Agilent Technologies, Berlin, Germany) gas chromatograph (GC) with a flame ionisation detector (FID). The methylation step and GC-FID analysis were performed as described in detail by Kristinova *et al*. (*17*). An internal standard 21:0 methyl ester (purity 99%; Nu-Chek Prep Inc., Elysian, MN, USA) was added to the extracted sample prior to methylation. Fatty acid methyl esters were identified by the comparison of their retention times with those of a reference solution (Nu-Chek Prep Inc.) analyzed under identical gas chromatographic conditions. The results were expressed as mass fraction (%) of each FA in the total FA mass (g FA per g lipid) and recalculated to give mg FA in 100 g product. Two replicates were run for each sample.

### NMR metabolomics

Fish fillet samples from both haddock and mackerel, and samples from the inner part of the fish cakes (*N*=3 from each group) were taken immediately after preparing the cakes and frozen at –80 °C. Low-molecular-mass metabolites were extracted from the frozen samples by using methanol, chloroform and water. This is a method commonly used for lipid extraction, but is also proposed for extraction of water-soluble metabolites in NMR studies, and it is particularly found suitable for tissues with high lipid content. The volumes and methodology were used according to Bligh and Dyer (*12*), but scaled down by a factor of 100, and using an average water content of circa 70% in the samples. Approximately 1 g of sample was extracted in triplicate for each type of fillet or fish cake. The resulting water/methanol phase was evaporated in a vacuum centrifuge (Eppendorf^TM^ Concentrator plus, Oslo, Norway) at 30 °C for 1 h, freeze dried, and dissolved in 550 µL phosphate-buffered saline (PBS, pH=7.4).and transferred to 5 mm NMR tubes. The PBS buffer was made using deuterium oxide (99.8% D; Sigma-Aldrich, Merck, Berlin, Germany) with 4,4-dimethyl-4-silapentane-1-sulfonic acid (DSS)-d6 (98% D; Sigma-Aldrich, Merck, Berlin) as an internal standard (0.5 mM). NMR spectra were recorded on a Bruker Avance 600 MHz spectrometer (Bruker BioSpin GmbH, Rheinstetten, Germany) at 25 °C with a cryo-probe operating at a ^1^H frequency of 600.182 MHz (at the NMR laboratory at the Faculty of Natural Sciences, Norwegian University of Science and Technology, Trondheim, Norway). The ^1^H NMR spectra were obtained using the Bruker pulse sequence noesygppr1d (1D NOESY pulse sequence with presaturation for water suppression). The following settings were used: sweep width 20 ppm, time domain 64k data points, acquisition time 4.1 s, relaxation delay 1.0 s, number of scans 128, dummy scans 4. The raw data were multiplied with a 0.5 Hz exponential line broadening factor before zero filling (real spectrum size (si) 64k data points) and Fourier transformed. Low-molecular-mass metabolites in the resulting spectra were identified and quantified by computer-assisted manual ﬁtting with Chenomx NMR Suite (v. 8.4, Chenomx, Edmonton, Canada). The results were expressed in mg/100 g of fish cake, or in mM of the extracts for calculation of the freshness indicator K-value, as defined by Saito *et al*. (*18*). The K-value is defined as the ratio of the sum of inosine (Ino) and hypoxanthine (Hx) to the sum of all adenosine triphosphate (ATP) related catabolites. In the current study, the K-value of the raw material was calculated as the molar ratio of Ino and Hx *vs* the sum of inosine monophosphate (IMP), Ino and Hx (since no ATP, adenosine diphosphate or adenosine monophosphate were detected). The K-value was also calculated for the prepared fish cake to evaluate if there were significant changes in the taste compounds IMP and Hx during cooking.

### Colour parameters

Colour parameters of haddock and mackerel fish cakes were measured instrumentally using a Minolta Chroma meter CR-400 (Konica-Minolta, Osaka, Japan). Before starting the analysis, the instrument was calibrated with a standard white plate. The data were recorded in colour coordinates of L* (lightness, black=0, white=100), a* (redness>0, greenness<0), and b* (yellowness>0, blueness<0) according to the Commission Internationale de l’Éclairage (CIE) Lab scale. The measurements were performed on three preselected locations of the inner surface of each fish cake at room temperature, six readings were conducted per each fish cake and the average was calculated.

### Texture parameters of fish cakes

Texture profile analysis (TPA) was conducted to measure the hardness and cohesiveness of the fish cakes. The analysis was performed at room temperature ((20±5) °C), with a texture analyzer (SMS Stable Micro Systems, Ltd., Surrey, UK) equipped with a 5-kg load cell according to the method described by Hultmann and Rustad (*19*). Prior to analysis, each fish cake was cut in two parts using a kitchen knife, and the measurements were performed on the inner part of the fish cake section. A flat-ended aluminum cylinder of 35 mm in diameter was pressed into the inner part of the fish cake at a constant speed of 1 mm/s until it reached 60% of its height. The holding time between the compressions was 5 s. The maximum resistance force was recorded in Newton (N) and expressed as the average of two determinations per fish cake. Cohesiveness, which represents the force holding the integrity of fish cake structure while preventing it from rupturing, was calculated as the ratio of areas delimited by the curves of the second and the first compression of each separate fish cake section.

### Microbiological analysis

Microbiological analysis of fish cakes was performed by an accredited lab (Analysesenteret in Trondheim, Norway). The total aerobic plate count, thermotolerant coliform bacteria and anaerobic sulphite-reducing bacterial counts were analysed using the following methods: for total aerobic plate count NMKL method 86 (*20*), for thermotolerant coliform bacteria an internal method of the accredited lab, and for anaerobic sulphite-reducing bacteria NMKN method 56 (*21*). The samples were prepared as described by NMKL method 91 (*22*). Briefly, a 1- to 2-mm thick piece of surface was aseptically cut using a 10 cm^2^ template and transferred to a Stomacher filter bag. Saline peptone diluent (0.85% NaCl, 0.10% peptone, 9 mL) was added and the sample was homogenized at 230 rpm. The homogenate was defined as dilution 10:1. Dilution series of the homogenate were made in test tubes with saline. The same samples were used both for total plate count and iron agar count. Total plate count analysis was performed according to NMKL 86 (*20*).

### Sensory analysis

Sensory tests of fish cakes were conducted in Norway by a group of 25 consumers (*N*=25). The selection was based on the availability of the respondents, with the use of two parities: about 50% women/men and about 50% people aged 55+. At the sampling stage, the following groups were excluded from the tests: people with food allergies, people who do not eat fish or fish products and people participating in other consumer tests 3 months prior to the survey. Despite the random selection of respondents, the study cannot be considered as a survey conducted on a representative nationwide sample of respondents (*23*), but as a pilot study.

Fish cake samples were warmed up for 2 min at 650 W in a microwave oven (model NF4046; Electrolux, Stockholm, Sweden) before the sensory test. Samples were coded with random numbers and given in a random order. In order to avoid tiredness of the senses, the number of tested products was limited to 3 (fish cakes with different formulations). The respondents described the feeling using sensory descriptors and the evaluations were recorded using a tablet mobile device with a dedicated questionnaire uploaded.

The subject of the tests were sensory aspects of the discussed products in three assessment phases (*23*): (*i*) basic sensory characteristics, (*ii*) satisfaction with individual sensory elements, and (*iii*) overall satisfaction and the willingness to purchase the product.

Basic sensory characteristics described the perception of selected descriptors of the product experienced during consumption (*24*). The intensity of each basic sensory descriptor was assessed on a structured graphical scale with boundary markings, corresponding to 0-10 units: for the saltiness 0 meant ’too little salt‘ and 10 ’very salty‘, for the firmness ’0‘ meant ’very soft’ and 10’ ’very hard’, for the juiciness 0 meant ’very dry’ and 10 ’very juicy’, for the intensity of the aroma 0 meant ’not very intense, faintly perceptible’ and 10 ‘very intense’.

The research on the satisfaction with individual sensory elements was based on asking the respondents to rate selected product features (meat structure rating, meat colour rating, aroma rating) on a descriptive 8-point scale (*25*), where 1 was unacceptable and 8 was excellent (intermediate grades were also described to respondents).

The level of overall satisfaction with the product and the willingness to purchase it was assessed on the 5-point Likert scale (where 1 was very negative, 3 was neutral, and 5 was very positive). The acceptability index was calculated by summing up the values of the answers to the questions regarding the level of satisfaction and willingness to purchase. Measurements of both questions were made on the five-point Likert scale, therefore the values of the indicator were in the range from 2 to 10 points. The calculated index was treated as an interval quantitative scale (*26*).

### Statistical analysis

Statistical analysis and data processing were conducted using Statgraphics Centurion XVI and Minitab 18 (*27*). Experimental data in the tables and figures are the average values of triplicate observations unless otherwise specified. Values marked with different letters are significantly different (p<0.05). Comparison of mean values was conducted using one-way ANOVA with Minitab 18 (*27*). For NMR data ANOVA with Tukey comparisons of means assuming equal variance was applied when normality and homogeneity of variance were confirmed by Anderson-Darling normality and Levene’s tests, respectively. For some metabolites, these conditions were not met, *e.g*. due to very different ranges in haddock and mackerel samples (*e.g*. for histidine), and in this case ANOVA was run as above on the fish cake samples only, while a two-sample *t*-test was performed on the raw material (haddock and mackerel). Also, for metabolites with zero values in one or more of the raw materials (anserine, citrate), the ANOVA was run on the fish cake sample only.

For consumer acceptance and sensory test results, statistical analysis and data processing were conducted using the IBM SPSS Statistics v. 25 statistical package (*27*). Basic descriptive statistics, frequency statistics, normality tests of the Shapiro-Wilk distribution, univariate variance analysis, Pearson’s r correlation analysis and linear regression analysis with multiple predictors were calculated (*25*). Differences were considered significant at p<0.05.

## RESULTS AND DISCUSSION

### Lipid content and fatty acid composition

The lipid content in mackerel and haddock fillets used in the study was (22.1±0.5) and (0.7±0.1) %, respectively (Table 1). The frying in rapeseed oil increased the final lipid amount in *w*(haddock)=100% fish cakes to 2%. The mass fraction of polyunsaturated fatty acids was lower in the haddock fish cakes (*w*=(22.8±0.1) and (51.6±0.5) g/100 g total fatty acids, respectively) than in the haddock raw material.

**Table 1:** Lipid content and fatty acid composition of haddock and mackerel mince and fish cakes prepared thereof

Composition	Raw material		Fish cake		
*w*(mackerel)/%		
Mackerel	Haddock	0	25	50
*w*(total lipid)/(g/100 g)	22.1±0.5	0.7±0.1	2.0±0.1	6.5±0.1	9.9±0.1
*w*(fatty acid)/(mg/100 g):					
saturated	(25.3±0.4)^c^	(29.5±0.4)^b^	(36.2±0.1)^a^	(29.1±0.1)^b^	(28.6±1.5)^b^
monounsaturated	(45.7±0.6)^a^	(18.9±0.2)^d^	(41.1±0.1)^c^	(43.2±0.1)^b^	(45.0±0.8)^a,b^
polyunsaturated	(29.0±0.2)^b^	(51.6±0.5)^a^	(22.8±0.1)^d^	(27.7±0.1)^b,c^	(26.4±0.7)^c^
*w*(EPA+DHA)/(mg/100 g)	(3890±16)^a^	(199±3)^d^	(183±3)^d^	(943±18)^c^	(1296±135)^b^

The same letter in each column determines no statistical difference among the values of fish cakes. Different letters denote significant differences in ANOVA

Based on the fatty acid composition of the fish cakes (Table 1), the recommended daily intake of 250 mg of EPA and DHA (*28*) can easily be reached by consumption of fish cakes enriched with mackerel. By consuming circa 200 g of fish cakes with 25% mackerel (or 150 g of the fish cake with 50% mackerel), the recommended amount of EPA and DHA for one week is reached.

### Lipid oxidation

Peroxide value is used as a quality parameter of an oil- or lipid-containing food product, although it is not directly related to its sensory quality (*29*). At PV>20 meq/kg (10 mmol/kg), the level of peroxides that must be formed to produce noticeable oxidative rancidity is reached; however, for some products this limit is lower (*e.g.* 10 meq/kg or 5 mmol/kg) (*29*). Peroxide value in the lipids extracted from the fish cakes was (4.3±0.6), (7.1±0.6) and (7.6±0.6) meq/kg or (2.2±0.3), (3.6±0.3) and (3.8±0.3) mmol/kg for fish cakes with haddock/mackerel ratios 100:0, 75:25 and 50:50, respectively. Thus, the oxidative quality of the lipids in fish cakes was acceptable. Recalculating the peroxide value per 1 kg product (Table 2), it is clear that the content of peroxides in the fish cakes was very low and measured in µeq (μmol) scale. The peroxide amount increased significantly (p<0.05) by increasing the mass fraction of mackerel in the fish cakes because of the increased amount of lipids per 100 g of product and because of the higher unsaturation in the fish cake with mackerel lipids (Table 2).

**Table 2:** Physicochemical characteristics of fish cakes prepared from mackerel and haddock minces

*w*(mackerel in fish cake)/%	Peroxide value		*w*(TBARS)_1_/(mg/kg)	*w*(TBARS)_2_/(mg/kg)	L*	a*	b*	Hardness/N	Cohesiveness
μeq/kg	µmol/kg
0	(87±11)^c^	(43.5±5.5)^c^	(0.2±0.0)^c^	(0.2±0.1)^c^	(81.2±0.4)^a^	(-2.2±0.1)^a^	(9.0±0.2)^a^	(99.3±12.4)^a^	(0.6±0.3)^a^
25	(465±38)^b^	(232.5±19.0)^b^	(0.6±0.0)^b^	(2.7±0.3)^b^	(77.5±0.5)^a,b^	(-1.4±0.2)^b^	(10.6±0.3)^a, b^	(82.1±11.3)^b^	(0.5±0.3)^a,b^
50	(786±49)^a^	(394.5±24.5)^a^	(0.8±0.1)^a^	(3.5±0.1)^a^	(73.9±0.6)^b,c^	(-0.8±0.1)^c^	(11.6±0.2)^b,c^	(70.5±5.4)^c^	(0.4±0.1)^b,c^

The same letter in each column denotes no statistical difference among the values of fish cakes. Different letters denote significant differences in ANOVA. *w*(TBARS)_1_=thiobarbituric acid reactive substances in methanol/water phase, *w*(TBARS)_2_=thiobarbituric acid reactive substances in chloroform phase, L* value=lightness, a*=redness and b*=yellowness

The TBARS limit for quality and acceptability of oils for human consumption is suggested on malonaldehyde (MDA) basis to be 7-8 mg/kg (*30*, *31*), but the ’acceptable’ value can also depend on the method used for both extraction and determination. Both lipid- and water-soluble TBARS were measured in fish cakes. The amount of lipid/chloroform soluble aldehydes was slightly higher than of methanol/water soluble aldehydes, even though the measured mass fractions were lower than the proposed ’acceptable’ value of MDA 8 mg/kg. One explanation for the higher amount of TBARS in the lipid/chloroform phase than in the methanol/water phase can be that the oxidation process in the samples is slow and triglyceride molecule are not cleaved to the low molecular hydrophilic oxidation compounds present in more oxidized lipids.

In previous studies on minced fish products, different TBARS mass fractions expressed as MDA have been reported, *e.g.*: fried whiting balls *w*<0.27 mg/kg (*32*), anchovy cakes *w*=0.9 mg/kg (*33*), rainbow trout fish burgers *w*<0.33 mg/kg (*34*). The mass fraction of TBARS in haddock and mackerel fish cakes also increased with increasing mass fraction of mackerel in the fish mass, which is, as already mentioned, due to the increased amount of lipids and unsaturated fatty acids (Table 2).

### Colour parameters of fish cakes

Colour parameters of haddock and mackerel fish cakes are given in Table 2. The lightness (L* value) of the fish cakes increased significantly (p<0.05) with increased mass fraction of haddock mince. This tendency can be explained by the lower mass fraction of dark muscle of haddock than of Atlantic mackerel. The significant (p<0.05) differences in redness (a* value) between fish cakes prepared from haddock mince alone, and fish cakes containing both haddock and mackerel mince in their formulations can be explained by the amount of myoglobin in these fish species (*35*, *36*). Myoglobin is the predominant pigment in dark muscle of Atlantic mackerel, which is found in very small amounts in haddock muscle. Differences in yellowness (b* value) between haddock and haddock/mackerel fish cakes can be explained by the differences in lipid oxidation occurring during the product preparation process (*35*). The high mass fraction of polyunsaturated fatty acids in Atlantic mackerel compared to haddock gives a higher susceptibility to lipid oxidation under elevated cooking temperatures. In accordance with PV and TBARS data, the fish cakes containing 25 and 50% mackerel mince underwent faster lipid oxidation. For all fish cake samples, the b* values increased towards a more yellow colour following the increase in lipid oxidation. This is in good agreement with a previous study of Cropotova *et al*. (*36*) showing that accumulation of secondary lipid oxidation products correlates with increased yellowness of fish cakes. This is supported by a significant correlation between the b* value and the amount of water-soluble TBARS (r=0.990, p<0.05) and fat-soluble TBARS (r=0.976, p<0.05).

### Texture parameters of fish cakes

Fish cakes prepared with higher mass fraction of haddock mince in the formulations had significantly (p<0.05) higher hardness and cohesiveness values than the fish cakes prepared with equal amounts of haddock and mackerel mince in the formulations, as shown in Table 2. This can probably be explained by better gelation properties of fish mince prepared with higher mass fraction of haddock with less fat. Saldaña *et al.* (*37*) found that instrumental texture properties such as hardness and cohesiveness were significantly affected by the fat content in the product formulation.

### Microbiological parameters of fish cakes

Total aerobic plate counts, the bacterial counts of thermotolerant coliform bacteria and anaerobic sulphite-reducing bacteria counts were *N*<10 CFU/g in fish cakes with haddock/mackerel mass ratio 100:0, 75:25 and 50:50%. Total aerobic plate count did not exceed the maximum limits (*N*=6 log CFU/g) of microbiological criteria for fish products (fish sticks, fish portions, fish cakes) given by International Commission on Microbiological Specifications for Foods (*38*).

### Sensory parameters

One of the main aims of the consumer testing was to define the acceptance of the novel fish cake prototypes (with mackerel replacing haddock). The assessment of product acceptance is based on the acceptability index, including the level of satisfaction with the product and the willingness to purchase it. Mean acceptability index for the white fish cakes (with 100% haddock, which are equivalent of the products available on the market) was 5.56 (on a 10-point scale). For novel fish cake prototypes the acceptability index was higher: 5.60 for fish cakes with 25% mackerel and 5.80 for fish cakes with 50% mackerel (data not shown).

The result of the one-way ANOVA analysis for the three fish cakes tested was not statistically significant. There were no important differences at the level of acceptability between novel fish cake prototypes and white fish cakes.

Frequency statistics of acceptability index is a confirmation of the existence of a market niche for newly developed fish cakes (where mackerel partially replaces white fish). We decided to take the value 8 for the acceptability index as a limit value showing the existence of any market potential for the product. It corresponds to definite fulfillment of expectations or decisive willingness to buy, or simultaneous moderate willingness to buy at a moderate level of satisfaction. With this restrictive value of consumer acceptability, the white fish cakes were accepted in the survey by 28% of consumers. Percentage of consumers that accepted novel fish cake prototypes was even higher: 36% for fish cakes with 25% mackerel and 32% for fish cakes with 50% mackerel (Table 3).

**Table 3:** Frequency statistics for the acceptability index of individual products

*w*(mackerel in fish cake)/%	Point range	Acceptability index	
*N*	%
0	2–3	5	20.0
	4–5	7	28.0
	6–7	6	24.0
	**8 – 10**	**7**	**28.0**
50	2 – 3	6	24.0
	4 – 5	5	20.0
	6 – 7	6	24.0
	**8 – 10**	**8**	**32.0**
25	2 – 3	3	12.0
	4 – 5	11	44.0
	6 – 7	2	8.0
	**8 – 10**	**9**	**36.0**

Next, it was verified whether the level of acceptability is significantly related to the age of the respondent. The differences in the level of acceptability ratio between three age groups (18-30, 31-54 and 55+) for particular products were checked using one-way analysis of variance. The results were only statistically significant for fish cakes with 50% mackerel (Table 4).

**Table 4:** One-way ANOVA test for the acceptability index of individual products between the three age groups

*w*(mackerel in fish cake)/%	Age group			F-value	df	P-value	η^2^
18-30	31-54	55+
Mean		
0	4.2±2.4	5.7±2.4	5.9±1.8	1.53	2, 17.70	0.244	0.07
25	5.0±1.3	5.3±2.3	6.0±2.3	1.14	2, 25.32	0.336	0.03
50	7.0±1.5	4.9±2.3	6.1±2.4	3.76*	2, 23.74	**0.038**	**0.11**

df=degrees of freedom, P-value=level of marginal significance within a statistical hypothesis test, η^2^=proportion of variance accounted for by a factor

To determine the relationship among the sensory characteristics of the products, component acceptance and final acceptance index, a series of correlation analyzes was performed using the Pearson’s r ratio among the following variables: acceptability index, colour, acceptance, aroma acceptance, aroma intensity, saltiness, firmness, juiciness. Significant, high correlation (r=0.61) between juiciness and consumer acceptance is obvious and expresses the consumers' expectation that the fish cakes should be juicy. The high negative correlation (r=-0.55) between juiciness and firmness is also expected; firm products are usually perceived as less juicy. The high correlation between the acceptance of different components (of the aroma, meat structure and meat colour: r=0.47-0.49) and the total acceptability confirm that the assessment of the aroma and appearance are important elements of the product's evaluation. It should be noted that all tested products have a delicate aroma, so weak positive correlation between the intensity of the aroma and its acceptability (r=0.26) is not surprising (Table 5).

**Table 5:** Analysis of Pearson's correlation between sensory characteristics and the acceptability index of fish cakes

Variable	1	2	3	4	5	6	7
**Acceptance parameter:**							
1. Acceptability index	---						
2. Meat structure	0.47***	---					
3. Meat colour	0.45***	0.60***	---				
4. Aroma acceptability	0.49***	0.52***	0.56***	---			
**Basic sensory descriptor:**							
5. Aroma intensity	0.26**	0.18*	0.14	0.26**	---		
6. Saltiness	0.14*	0.02	0.05	0.13	0.30***	---	
7. Firmness	-0.23**	-0.15*	0.02	-0.03	-0.18*	0.35***	---
8. Juiciness	0.61***	0.34***	0.23**	0.17*	0.30***	0.36***	-0.55***

0.05<p<0.10, *p<0.05, **p<0.01, ***p<0.001

At the last stage, a linear regression model was created to select the best predictors of the acceptability value for fish cakes. Sensory attributes of products whose correlation values with the acceptability ratio of fish cakes were statistically significant (colour and aroma assessment and intensity, salt content, and juiciness) were used as predictors.

The created model was statistically significant, and the value of the adjusted R^2^ coefficient was 0.64 (Table 6), which means that approx. 64% of the variance in the acceptability ratio can be explained by the created model and its predictors.

**Table 6:** Coefficients of a linear regression model predicting the acceptability index values of fish cakes based on sensory descriptors and component acceptability

Model	Predicator	B	SE	β	t	F	df		p		R^2^	
Acceptability index	Constant	-0.72	0.95		-0.76	35.95		7.132		<0.001		0.64
	Meat structure	-0.02	0.11	-0.01	-0.17							
	Meat colour	0.28	0.10	**0.21**	2.71**							
	Aroma acceptability	0.48	0.10	**0.31**	4.74***							
	Aroma intensity	0.07	0.07	0.06	1.08							
	Saltiness	-0.33	0.08	**-0.25**	-4.14***							
	Firmness	0.05	0.08	0.04	0.64							
	Juiciness	0.68	0.08	**0.60**	9.01***							

**p<0.01, ***p<0.001. Constant=y-intercept, B=unstandardized beta, SE=standard error; β=standardized B coefficients; t=*t* test, F=F-value, df=degrees of freedom, p=p-value (test probability), R^2^=coefficient of determination

Statistically significant predictors in the final model were juiciness (β=0.60), aroma acceptability (β=0.31), saltiness (β=-0.25) and meat colour (β=0.21) (Table 6). This means that consumers especially expected fish cakes with high juiciness, while it is also important to produce fish cakes with the right (pleasant) aroma and right (probably not too dark) meat colour, and not too high salt content.

### Low-molecular-mass metabolites analyzed by HR NMR

Table 7 gives an overview of low-molecular-mass metabolites quantified in the muscle and fish cake extracts, and includes free amino acids, peptides and other small molecules such as nucleotide derivatives, organic acids and sugars. This pool of metabolites includes both taste-active components, freshness indicators and metabolites that are considered bioactive (*39*-*42*). For example, taurine is well recognized as beneficial for the cardiovascular system (*2*), and anserine is known as an antioxidant and is suggested to impact cognitive functions (*4*).

**Table 7:** Low-molecular-mass metabolites quantified in raw material and prepared fish cakes with different levels of haddock and mackerel

*w*/(mg/100 g)	Raw material		Fish cake		
*w*(mackerel)/%		
Haddock	Mackerel	0	25	50
Acetate	(1.7±0.5)^a^	(1.2±0.1)^a^	(2.3±1.1)^a^	(2.0±0.1)^a^	(2.1±0.1)^a^
Alanine	(14.9±1.2)^c^	(28.6±2.5)^a^	(12.0±2.2)^c^	(15.7±0.2)^bc^	(19.8±1.7)^b^
Anserine	(236±36)	n.d.	(146±14)^a#^	(122±78)^a#^	(91±7^)b#^
Citrate	n.d.	n.d.	(44±1.6)^a#^	(44.1±2.4)^a#^	(39.6±4.0)^a#^
Creatine	(506±50)^a^	(427±54)^ab^	(346±12)^bc^	(328±18)^c^	(323±22)^c^
Creatinine	(0.1±0.1)^b^	(0.5±0.9)^b^	(8.2±2.1)^a^	(7.5±1.4)^a^	(10.4±1.8)^a^
Dimethylamine	(1.9±0.5)^a^	(1.1±0.2)^b^	(2.0±0.1)^a^	(2.5±0.2)^a^	(2.1±0.3)^a^
Formate	(0.4±0.2)^ab^	(0.2±0.1)^b^	(0.6±0.1)^a^	(0.5±0.04)^a^	(0.53±0.02)^a^
Glutamate	(8.7±1.4)^d^	(46.7±5.4)^a^	(12.5±2.0)^cd^	(16.6±0.5)^bc^	(22.7±2.1)^b^
Glycerol	(3.9±2.2)^b^	(261±3)^a^	*	*	*
Glycine	(12.1±1.7)^a^	(12.7±0.8)^a^	*	*	*
Histamine	n.d.	n.d.	n.d.	n.d.	n.d.
Histidine	(3.3±0.4)**	(365±114)**	(3.0±0.2)^c#^	(66±2)^b#^	(105±18)^a#^
Hypoxanthine	(3.0±1.2)^b^	(6±0.4)^a^	(1.6±0.2)^b^	(2.9±0.3)^d^	(2.5±1.3)^b^
IMP	(134±27)^a^	(74±34)^b^	(66±2)^b^	(73±23)^b^	(58±4)^b^
Imidazole	(6.9±4.0)^a^	(1.1±1.0)^b^	(4.9±1.2)^ab^	(3.8±0.1)^ab^	(2.7±0.6)^ab^
Inosine	(62±25)^ab^	(90±1)^a^	(54±3)^b^	(43±2)^b^	(59±2)^b^
Isoleucine	(1.9±0.3)^c^	(4.9±0.1)^a^	(1.8±0.4)^c^	(2.40±0.03)^c^	(3.3±0.4)^b^
Lactate	(387±30)^ab^	(415±87)^a^	(273±4)^c^	(303±4)^bc^	(315±6)^abc^
Lactose	n.d.	n.d.	(1203±58)^a^	(1325±160)^a^	(1177±70)^a^
Leucine	(3.6±0.34)^c^	(9.8±1.3)^a^	(3.0±1.3)^c^	(5±0.1)^bc^	(7.2±0.5)^b^
Lysine	(7.6±0.6)^cd^	(33±2.7)^a^	(3.8±1.0)^d^	(11.0±0.2)^bc^	(14.4±4.1)^b^
Methionine	(1.9±0.5)^bc^	(4.4±0.1)^a^	(1.3±0.1)^c^	(2.3±0.1)^b^	(2.3±0.4)^b^
Phenylalanine	(1.4±0.6)^c^	(5.1±0.6)^a^	(1.4±0.6)^c^	(2.24±0.04)^bc^	(3.3±0.4)^b^
Succinate	(0.2±0.2)^c^	(0.17±0.006)^c^	(0.69±0.02)^b^	(0.93±0.01)^ab^	(1.2±0.1)^a^
Taurine	(63±5)^ab^	(72±6)^a^	(50±3)^c^	(60±1)^abc^	(56±7)^bc^
Trimethylamine	(0.1±0.1)^c^	(0.9±0.3)^b^	(0.7±0.1)^bc^	(1.5±0.4)^a^	(2.1±0.2)^a^
TMAO	(353±38)^a^	(138±18)^d^	(256±8)^b^	(215±9)^bc^	(180±11)^cd^
Tyrosine	(2.0±0.7)^cd^	(8.0±0.9)^a^	(1.2±0.1)^d^	(2.8±0.1)^bc^	(4.1±0.23)^b^
Valine	(3.2±0.4)^c^	(9.5±0.9)^a^	(3.0±0.9)^c^	(4.36±0.05)^bc^	(5.9±0.5)^b^
β-Alanine	(6.9±2.0)^a^	n.d.	(7.0±0.5)^a^	(4.9±0.1)^ab^	(4.0±0.4)^b^

Data are expressed in mg/100 g on wet mass basis (average with standard deviations, *N*=3). Different letters denote significant differences in ANOVA. For some metabolites, ANOVA was run only on the different fish cake samples, and these are denoted with #. **For histidine, the *t*-test showed differences in mean values of histidine for haddock and mackerel raw materials. n.d.=not determined

The most obvious difference in the profile of low-molecular-mass metabolites in muscle extracts from haddock and mackerel is the different levels of anserine and histidine (Table 7). Anserine is abundant in haddock (*w*=(236±36) mg/100 g), and not detected in mackerel, while histidine is dominant in mackerel (*w*=(365±114) mg/100 g) and not detected in haddock. Imidazole compounds, such as histidine and anserine, act as pH buffers in fish muscle; and active migratory fish species, such as mackerel, have high levels of free histidine (*42*). The level of histidine in mackerel is also relevant for food safety, since it is a precursor of histamine, which is responsible for scombroid poisoning caused by spoiled fish (with histamine above *w*=5-10 mg/100 g). However, in the present study, the histamine content in mackerel was below the detection limit (*w*<1 mg/100 g), confirming that the mackerel was of good freshness and had been treated hygienically.

The trimethylamine N-oxide (TMAO) mass fractions are in general higher in haddock (*w*=(353±38) mg/100 g) than in the mackerel (*w*=(138±18) mg/100 g). TMAO is known to be a significant contributor to the pool of cellular metabolites in whitefish, while pelagic fish are reported to have lower mass fractions of TMAO (*43*). The TMAO mass fractions are in agreement with previous studies on whitefish (cod *w*=340-380 mg/100 g) (*43*) and mackerel (*40*) (*w*=(130-170) mg/100 g). For teleosts (bony fish), TMAO are regarded as important for counteracting destabilization of proteins by pressure, and the content increases in deep waters (*43*). It has been observed that TMAO may have a protective effect in prion diseases, but TMAO has also been associated with adverse cardiovascular events, and its role as a potential biomarker or new therapeutic target has recently been reviewed (*44*, *45*). As previously shown in studies of fish sauce (*46*), free amino acids such as glutamate, pyroglutamate and alanine have been shown to be major contributors to fish-like taste and to its umami, sweetness and overall taste. Mackerel in general has higher levels of many free amino acids relevant for taste, such as alanine and glutamate, but especially histidine, than haddock.

The above-mentioned differences in low-molecular-mass metabolites in haddock and mackerel muscle are reflected in the corresponding profile of the fish cakes with different mass fractions of mackerel. It was observed that the standard deviation in the quantified metabolites was larger for the muscle samples than for the fish cake samples; it is proposed that the well-known variation within biochemical composition of fish individuals (due to factors such as age, size and gender) is evened out in the preparation of fish mince during the preparation of the fish cakes. For the fish cakes, lactose peaks (from the added milk) are dominant in part of the spectra, making it challenging to quantify glycerol and glycine due to overlapping peaks. In addition to the lactose, there was a clear increase in citrate, creatinine and succinate in the fish cakes compared to the raw material, which is also due to the added milk (*46*).

The trimethylamine (TMA) mass fractions increased from the fish raw material to the prepared fish cakes. TMA may be formed from TMAO, by certain bacteria during storage of fish and is responsible for a fishy odour (*42*). Different ranges of quality limits for TMA have been proposed, as summarized by Barbuzzi *et al*. (*47*), who chose an intermediate limit of *w*=6 mg/100 g in a study of fish mince. However, TMA is also reported to be present in milk (*48*). The TMA mass fraction in the haddock raw material was *w*=0.1 mg/100 g. In the fish cake with haddock, the mass fraction of TMA was *w*=0.7 mg/100 g even though the haddock mass fraction was only approx.. 74% in the fish cake (22% was milk). The TMA also increased with increasing addition of mackerel in the fish cake (to *w*=2.1 mg/100 g in the fish cake with 50% mackerel). This increase is higher than the theoretical increase due to the addition of mackerel, and the volume of added milk was constant. It is therefore proposed that even though some TMA is present in the milk, the increase in TMA mass fractions from raw material to fish cakes is probably due to microbial degradation of TMAO. The fish cake with haddock was prepared at the beginning of the day, while the cake with 50% mackerel some hours later, so in addition to increasing mackerel content (with highest TMA values), some TMA was also formed during the production day.

Compared to the traditional recipe (without the addition of mackerel), inclusion of mackerel significantly increased the mass fraction of His in the fish cakes. Ala and Glu increased somewhat but to a lesser content and the increase was significant in the fish cake with 50% mackerel. The higher mass fraction of such free amino acids in mackerel fish cakes can be one of the explanations of the sensory analysis showing that the aroma intensity of fish cakes with mackerel was higher than when using only haddock as raw material. However, since the taste is a function of many different taste compounds (*49*) (such as nucleotides, peptides, volatile compounds, and fatty acid derivatives), a further investigation of the correlation of the free amino acids and sensory attributes was beyond the scope of this topic.

The mass fraction of taurine was neither significantly different between haddock/mackerel nor between the three types of fish cakes: the mass fraction in mackerel (*w*=72 mg/100 g) was similar to the values found by Gormely *et al.* (*2*) (*w*=78 mg/100 g). The mass fraction of TMAO, anserine and β-alanine was reduced when mackerel was added to traditional fish cakes.

**Fig. 1:**
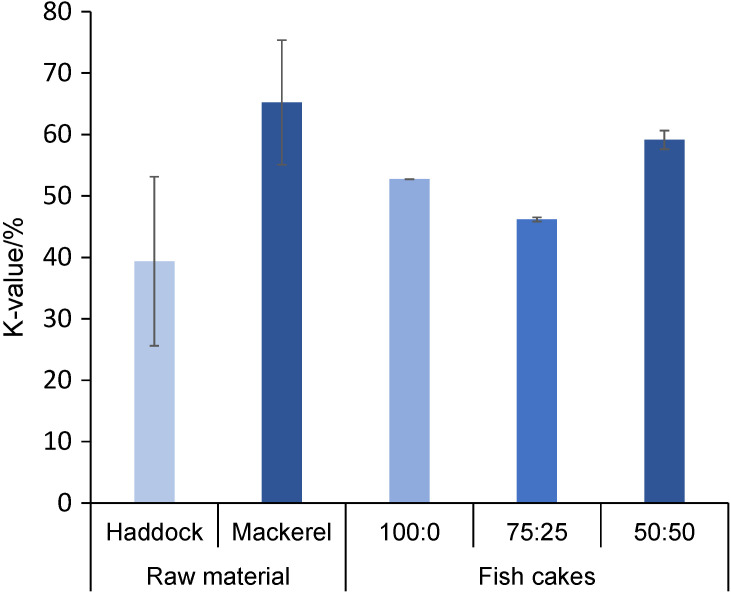
K-value (ratio of inosine and hypoxanthine to the sum of all adenosine triphosphate-related catabolites) for haddock and mackerel and fish cakes (FC) prepared thereof with different mass ratios of haddock and mackerel: 100:0, 75:25 and 50:50%

The acceptable K-values differ among fish species, but generally a K-value<20% is categorized as very fresh, while a K-value of 50-70% is regarded as moderately fresh. Even though the K-value was higher for the mackerel fillets than the haddock fillets (Fig. 1), both were of acceptable freshness (<70%), even though large variations were seen. The K-value did not increase significantly during preparation of haddock fish cakes, *i.e.* the K-value of haddock fillets was (40±14) %, while for the haddock fish cakes, the value was (53±0) % (Fig. 1). In addition, the K-values of fish cakes with haddock/mackerel mass ratio 75:25 and 50:50 (46 and 60%, respectively) was similar to the K-value of the raw material (46 and 52%, respectively). This implies that no significant breakdown of IMP to Ino or Hx took place during storage prior to cooking and during cooking. IMP is related to the umami fish taste of fresh seafood, while Hx is a contributor to the bitter off-flavour of spoiled fish and are thereby relevant sensory quality indicators also of the final product.

## CONCLUSIONS

Partial replacement of traditionally used haddock mince by mackerel mince in fish cake formulations significantly improved the nutritional profile of the product by increasing the EPA and DHA mass fractions. This resulted in softer and more tender texture due to higher fat mass fraction in fish cake. However, fish cakes enriched with mackerel mince had a significant increase in redness due to higher myoglobin content, and increased yellowness as a result of accumulation of secondary lipid oxidation products: water-soluble (r=0.990, p<0.05) and fat-soluble TBARS (r=0.976, p<0.05). Nevertheless, none of the fish cakes including the ones enriched with mackerel mince exceeded the limits of the oxidative lipid stability.

Sensory studies indicated that the increase in mackerel mass fraction positively affected the juiciness and aroma of fish cakes, while slightly negatively shaping the colour of the product and saltiness. These effects, in a way, offset each other, taking into account that the total rating of fish cakes with white fish and 25 and 50% replacement of mackerel is similar, and the differences are not statistically significant. Furthermore, 30 metabolites were quantified by using NMR metabolomics, and several of these are relevant for taste and nutritional value. The mass fraction of anserine, trimethylamine N-oxide and β-alanine decreased, while the mass fraction of histidine, glutamate, alanine increased with the addition of mackerel.

This study is based on the work supported by the JPI project ProHealth ’Innovative processing to preserve positive health effects in pelagic fish products’, RCN 259582/E50, funded by Norwegian Research Council, Oslo, Norway.
